# COVID-19 vaccine acceptance, hesitancy, and determinants among physicians in a university-based teaching hospital in Thailand

**DOI:** 10.1186/s12879-021-06863-5

**Published:** 2021-11-22

**Authors:** May Sirikalyanpaiboon, Krittin Ousirimaneechai, Jeerath Phannajit, Panyavee Pitisuttithum, Watsamon Jantarabenjakul, Roongruedee Chaiteerakij, Leilani Paitoonpong

**Affiliations:** 1 Department of Medicine, Faculty of Medicine, Chulalongkorn University, Bangkok, Thailand; 2 Division of General Internal Medicine, Department of Medicine, Faculty of Medicine, Chulalongkorn University, Bangkok, Thailand; 3 Division of Clinical Epidemiology, Department of Medicine, Faculty of Medicine, Chulalongkorn University, Bangkok, Thailand; 4 Division of Nephrology, Department of Medicine, Faculty of Medicine, Chulalongkorn University, Bangkok, Thailand; 5 Division of Gastroenterology, Department of Medicine, Faculty of Medicine, Chulalongkorn University, Bangkok, Thailand; 6 Division of Infectious Diseases, Department of Pediatrics, Chulalongkorn University, Bangkok, Thailand; 7 Center of Excellence for Innovation and Endoscopy in Gastrointestinal Oncology, Faculty of Medicine, Chulalongkorn University, Bangkok, Thailand; 8 Division of Infectious Diseases, Department of Mediciine, Chulalongkorn University, 1873 Rama IV Road, Patumwan, Bangkok, 10330 Thailand

**Keywords:** COVID-19, Vaccine hesitancy, Vaccine acceptance, SARS-CoV-2 vaccine, Healthcare, Physicians

## Abstract

**Background:**

The COVID-19 vaccines provide renewed hope in the fight against the recent pandemic. To ensure widespread vaccination, it is crucial to analyze vaccine willingness and its determinants among physicians, key health care influencers. This study aimed to assess acceptance rate and identify factors associated with vaccine hesitancy among Thai physicians.

**Methods:**

A cross-sectional online-based questionnaire was distributed to all physicians at King Chulalongkorn Memorial Hospital during March 31, 2021 to April 30, 2021 in order to assess their attitudes toward receiving the COVID-19 vaccine. Reasons for vaccine acceptance and refusal as well as predictors of vaccine hesitancy were analyzed by bivariate and multivariable analysis.

**Results:**

A total of 705 complete responses were received with 95.6% (n = 675) of physicians expressing willingness to receive a COVID-19 vaccine. Only one of the 31 physicians (4.4%) who expressed a hesitancy or unwillingness to be vaccinated was a faculty member; the others were physicians-in-training. Approximately one-fifths of physicians surveyed were also not willing to recommend the vaccine to their family members (21.4%, n = 151) or patients (18.7%, n = 132). Using multivariable logistic regression, vaccine hesitancy was independently associated with preference for particular vaccines over the government allocated option, especially for mRNA vaccine (aOR 8.86; 95% CI 1.1–71.54; p = 0.041). Vaccine literacy showed an inverse relationship (aOR 0.34; 95% CI 0.13–0.9; p = 0.029) with vaccine hesitancy. Uncertainty of the vaccine efficacy (83.9%) and fear of adverse events (48.4%) were major concerns contributing to vaccine hesitancy.

**Conclusion:**

This study revealed a high rate of physician willingness to take the COVID-19 vaccine especially among staffs; however, a significant proportion would not currently suggest vaccination to their families or patients. Restrictions on vaccine choice and vaccine illiteracy, together with concerns over adverse effects and uncertainty of efficacy, were associated with negative attitudes toward vaccination. To raise acceptance of the vaccination program, efforts should be made to balance individual preference for vaccine type in addition to increasing the availability of accurate data on safety and efficacy for each vaccine.

**Supplementary Information:**

The online version contains supplementary material available at 10.1186/s12879-021-06863-5.

## Background

Since December 2019, the coronavirus disease 2019 (COVID-19), a disease caused by severe acute respiratory syndrome coronavirus 2 (SARS-CoV-2), has emerged as a pandemic outbreak affecting over hundreds million people around the world [1, 2]. Several prevention measures have been deployed in response to the alarming rate of infection spread and severity. For the past year, public attention has focused on the development and implementation of a vaccine that can serve as a reliable and cost-effective preventive tool to combat the disease [3]. More than 90 COVID-19 vaccines had been developed with 27 vaccines in clinical trials phase III and 15 vaccines approved for emergency use by at least one country [4]. In addition to vaccine safety, efficacy, and cost-effectiveness, public acceptance plays a significant role in measuring overall effectiveness [5].

Vaccine acceptance is defined by “the degree to which individuals accept, question, or refuse vaccination.” It is a determinant for vaccine uptake rate, and consequently vaccine distribution success [6]. Debates over the effectiveness and safety of vaccinations in general have gained momentum around the world, posing a serious challenge to global public health, according to WHO in 2019 [7]. COVID-19 vaccine acceptance rates have fluctuated from 23.6 to 97% [8–12] including rates between 27.7 and 78.1% from surveys among healthcare workers [13, 14]. The main determinants of vaccine acceptance found in those studies included regular vaccination against influenza and vaccine availability. Studies have found that vaccine hesitancy stems from safety concerns, especially potential long-term side effects. The acceptance of COVID-19 vaccination has also been shown to be influenced by demographic factors such as age, sex, marital status, and education level [8–14].

Several countries in Asia, increasingly affected by COVID-19 pandemic, are currently facing vaccine delivery challenges [1]. By March 2021, vaccines of various varieties became accessible in limited quantities due to high demands. Out of four vaccines listed in the WHO Emergency Use Listing (EUL), including ChAdOx1 nCoV-19, Ad26.COV2.S, BNT162b2 and mRNA-1273, the Thai vaccination agency only chose AstraZeneca/Oxford COVID-19 vaccine for the elderly and brought CoronaVac, an inactivated vaccine from a Chinese manufacturer with then limited data, for younger adults [15]. To make matters worse, public opinion on COVID-19 immunization continues to fluctuate as false information flows through various media channels and social media platforms.

The paucity of government-run vaccination options as well as the public outcry concerning the poor vaccine implementation plan could lead to a fall in the vaccine acceptance rate. Initial estimates in November 2020 have shown vaccine acceptance among general Thai population as high as 77–87% in online social media based surveys conducted by overseas organizations namely Yougov and the Johns Hopkins Center for Communication [16, 17]. However, no study was undertaken in Thailand and South-East Asian countries to specifically examine physicians’ or health-care workers’ attitudes toward the COVID-9 vaccine.

Since physicians and healthcare workers are seen as shepherds for public health and will be given priority for vaccinations, it is crucial to understand their attitudes toward COVID-19 vaccinations in order to help overcome obstacles to widespread vaccination [7]. Currently, little data has been collected and analyzed specifically on physician attitudes on vaccine acceptance and factors that play a role in their willingness which can influence to their patients and families/friends networks. This study aimed to fill this research gap and determine COVID-19 acceptance and predictors, as well as attitudes toward the government allocated COVID-19 vaccines, among physicians in Thailand.

## Study design and methods

### Design and sample

The study was approved by Institutional Review Board of the Faculty of Medicine, Chulalongkorn University, Bangkok, Thailand (IRB number 279/64). A cross-sectional study was conducted during March 31, 2021 to April 30, 2021 enrolling physicians currently working at the King Chulalongkorn Memorial Hospital, Bangkok, Thailand. A google-service based survey was distributed through links on social media outlets such as LINE, Instagram, and Facebook. The online-based survey was preferred to ensure timely, comprehensive, and high-yield data acquisition and analysis. We deployed a snowball sampling technique, a non-probability sampling method which yields a convenient sample, to recruit physician participants by distributing the questionnaires through various social media networks of each division and department in the hospital. Data from fully completed questionnaires was retrieved and statistical analysis was performed. A sample size of at least 315 complete responders would provide a confidence level of 95% with margin of error of 5% to estimate vaccine acceptance rate among a total of 1736 physicians on-duty at King Chulalongkorn Memorial Hospital at the time of survey assumed maximum standard deviation [18]. Informed consent was obtained from all subjects (none under the age of 18). Data were collected anonymously, and no personally identifying information was collected.

### Demographic data

General baseline data including age, gender, religion, personal health issue, role of physicians on medical teams (residency, fellowship and attending physicians), year in training and specialty were collected. Data on experience of providing direct care for COVID-19 patients and physician exposure to high aerosol conditions were stratified as regular, occasionally, and never occurring. Participants also reported prior receipt of an influenza vaccine (yearly, sometimes, or never).

### Study questionnaire and variable definitions

The questionnaire was adapted and simplified from previous studies [13, 14] to be self-administered and confidential to ensure maximum response and minimize potential biases. All questions were written in Thai since all physicians were native Thai speakers. English-translated version of the questionnaire is available in the Additional file 1: Appendix A. A 30-person pilot survey was implemented to refine question wording and to ensure a survey completion time of 5–10 min. The questionnaire was divided into five sections: demographic data, opinions on COVID-19 disease, vaccine acceptance/hesitancy, attitudes toward specified vaccines and COVID-19 pandemic-related information.

#### Opinions on COVID-19 disease

Using a five-point Likert scale ranging from strongly agree to strongly disagree, respondents were asked to select the option that best fit their opinion or belief. General perceptions were determined by the statements: “COVID-19 is a catastrophic disease”, “COVID-19 poses a significant threat to social and economic well-being”, and “COVID-19 is preventable.”

#### Vaccine acceptance/hesitancy

We asked physicians if they were planning to receive the vaccine once it became available with response options including definitely yes, uncertain, and definitely no. With the same response choice, respondents were also asked whether they would suggest vaccination to a family member or a patient. Factors related to willingness or reluctance toward receiving a COVID-19 vaccine were selected from prelisted choices (Additional file 1: Appendix A). Participants had the option of selecting more than one factor. Physicians were also asked to select the most concerning side effect of the vaccine from the same set of prelisted choices (Additional file 1: Appendix A).

#### Attitude toward specified vaccine

Using a five-point Likert scale ranging from strongly disagree to strongly agree, we measured agreement with a set of statements coverings vaccine perceptions and concerns. Participants were asked to respond to a set of statements for each of the vaccine types (inactivated virus, viral vector, and mRNA). Statements such as “You believe that this form of vaccine will prevent transmission, symptomatic disease, and death” were used to test efficacy perceptions. Concerns regarding COVID-19 vaccine safety were evaluated by responses to the following sentences: “You have confidence in vaccine safety in terms of serious or life-threatening side effects, general adverse reactions, and long-term safety.”

#### COVID-19 pandemic-related information

Participants were asked to rate the amount of information they obtained about COVID-19 and their level of self-perception awareness. We also asked about the sources of the information.

### Statistical analysis

Baseline characteristics of participants were presented as counts and percentages for categorical variables, while continuous variables were presented as mean (standard deviation) and median (interquartile range; IQR). General acceptance of a COVID-19 vaccine was our primary outcome; “no” and “uncertain” were combined to reflect vaccine hesitancy, while “definitely yes” reflected vaccine acceptance. Demographic differences between two groups were performed using a Mann–Whitney-U test for continuous variables due to non-normal distribution and Fisher’s exact test for categorical variables which is preferable to the chi-squared test because it is an exact test and valid for all sample size [19]. Participants’ responses on five-point Likert scales were shown as diverging stacked bar charts and further compared between acceptors and hesitators by using Mann–Whitney-U test. Shapiro–Wilk test for normality was used together with histogram to determine distribution of each variable.

Multivariable logistic regression was performed to assess the association of determinants with COVID-19 hesitancy. Candidate covariates included sex, age, presence of comorbidity, physician role (resident/fellow/staff), specialty (general/medical/surgical), experience of providing direct care for COVID-19 patients, exposure to high aerosol conditions, preferred vaccine type (inactivated/viral vector/mRNA), five-point Likert scale of attitude and information about COVID-19 (treat as continuous variable), and information sources (treat as dichotomous variable, yes or no). The linearity assumption of Likert scale covariates were check using “lincheck” function which plot the ln(odds) of being hesitators against each scale. Variables that demonstrated a possible association from bivariate analysis (p < 0.20) were entered into the multivariable model. Results were presented as adjusted odds ratios (OR) and 95% confidence intervals (95%CI). We further performed sensitivity analyses by adding known predictors which were shown association with vaccine acceptance from previous studies including age, gender, and presence of comorbidity [13, 14] and reducing the threshold of p-value to 0.1 and 0.05 during variable selection for the multivariable model. All analyses were conducted using STATA version 16 (StataCorp, College Station, Texas). The statistical significance level was set at two-sided alpha of 0.05 without correction of multiple testing.

## Results

### Baseline characteristics and demographic data

A total of 705 of 1736 (40.6%) physicians completed the survey. Participant characteristics are shown in Table 1. In brief, females represented 50.9% of the sample. Median age (IQR) was 30.0 (28.0–33.0). Majority (93.9%) were Buddhist. Having at least one comorbidity was reported by 129 (18.3%) respondents including allergic rhinitis (n = 48, 37.2%), followed by hypertension (n = 16, 12.4%) and dyslipidemia (n = 12, 9.3%). Participants included medical residents (n = 406, 57.6%), clinical fellows in training (n = 128, 18.2%) and faculty members (n = 171, 24.3%). Medical clerkship personnel (internal medicine and pediatric) were the most frequent specialty at 37.6%, followed by surgical clerkship personnel at 36.7%, and general practitioners at 25.7%. None reported being previously diagnosed with COVID-19 infection. Nearly half (47.3%) of physicians had directly cared for COVID-19 patients, while 45.8% reported working in high-aerosol environments. Annual influenza vaccine recipients accounted for 67.9% of respondents, 29.2% reported receiving vaccinations but not on an annual basis, while almost 3% had never received a flu vaccine.

**Table 1:** Characteristics of study participants

Variables	Statistics	
Number of participants	n	705
Age	Median (IQR)	30.0 (28.0–33.0)
Female	n (%)	359 (50.9%)
Religion^†^	n (%)	
Buddhism		662 (93.9%)
Christianity		21 (3.0%)
Islam		3 (0.4%)
Others		19 (2.7%)
Presence of comorbidity	n (%)	129 (18.3%)
Physician role^†^	n (%)	
Resident		406 (57.6%)
Fellow		128 (18.2%)
Staff		171 (24.3%)
Department^†^	n (%)	
General/Others		181 (25.7%)
Medical		265 (37.6%)
Surgical		259 (36.7%)
Direct care of COVID-19 patients	n (%)	333 (47.2%)
High aerosolization work settings	n (%)	322 (45.7%)
Previous influenza vaccination^†^	n (%)	
Never		20 (2.8%)
Sometimes		206 (29.2%)
Yearly		479 (67.9%)
Preferred vaccine type^†^	n (%)	
Inactivated		162 (23.0%)
Viral vector		122 (17.3%)
mRNA		251 (35.6%)
Any		170 (24.1%)
Information sources (yes or no)		
Articles/Scientific journal	n (%)	458 (65.0%)
TV/Newspaper	n (%)	249 (35.3%)
Social media	n (%)	473 (67.1%)
Hospital media	n (%)	295 (41.8%)
Academic conference	n (%)	196 (27.8%)
Friend/Family	n (%)	180 (25.5%)
Others	n (%)	1 (0.1%)
Willingness to recommend vaccine to family^†^	n (%)	
No		20 (2.8%)
Yes		554 (78.6%)
Not sure		131 (18.6%)
Willingness to recommend vaccine to patients^†^	n (%)	
No		14 (2.0%)
Yes		573 (81.3%)
Not sure		118 (16.7%)

*n (%)* number and percentage; *IQR* interquartile range

^†^One best choice question

### General attitude toward COVID-19

**Fig. 1:**
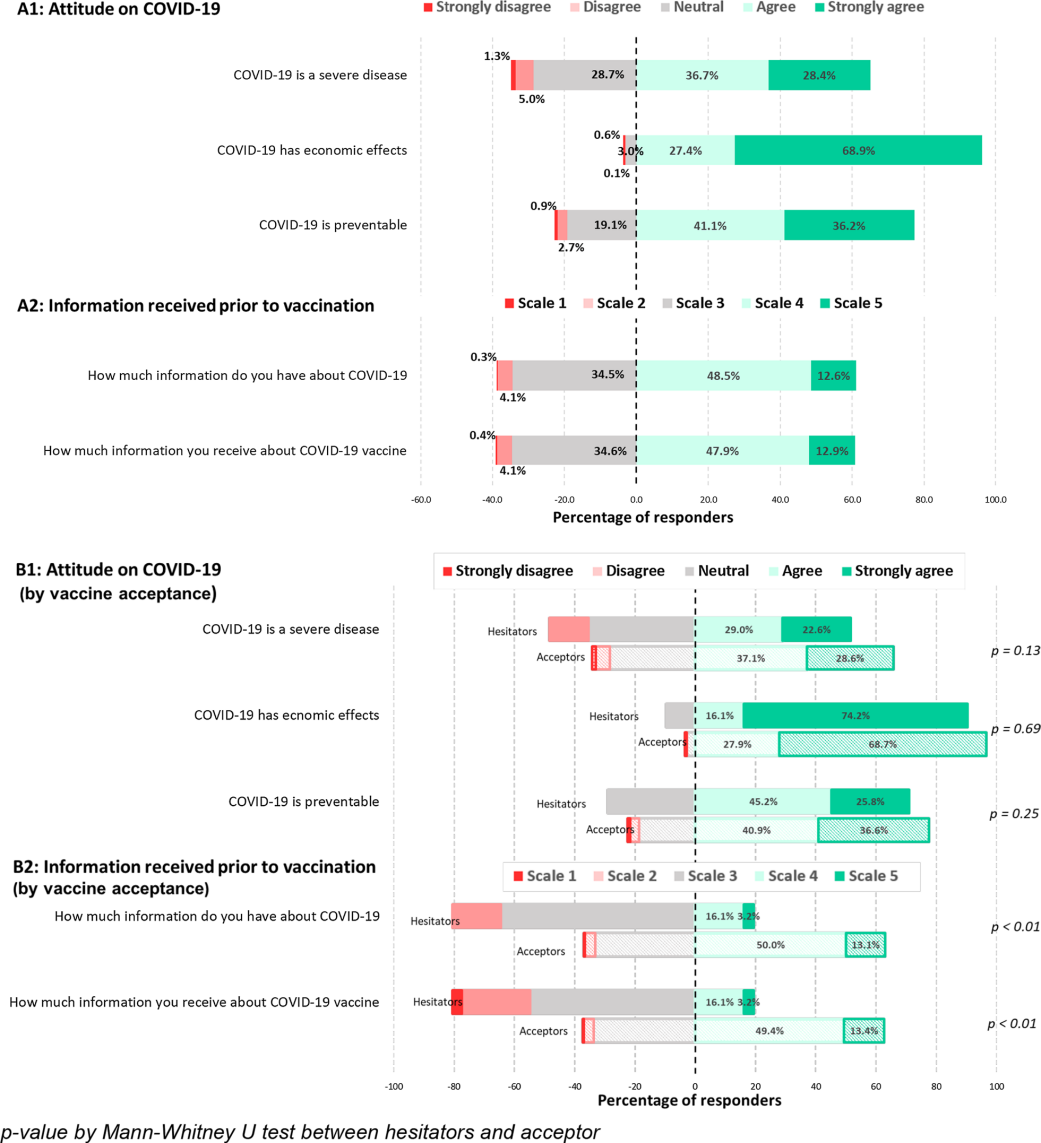
Distribution of responses regarding attitude and information received toward COVID-19 pandemic and COVID-19 vaccine

In our survey, most participants acknowledged (reported agree or strongly agree) that COVID-19 is a severe disease (65.1%) and that it is a preventable disease (77.3%). The negative economic effect of COVID-19 was nearly unanimously agreed (96.3%). Furthermore, 61.1% rated their understanding of the COVID-19 vaccine as “moderate to solid” with 60.8% believed that they had received sufficient information about the COVID-19 vaccine (Fig. 1).

### Vaccine acceptance

Among the 705 participants, a very high percentage of physicians (n = 675, 95.7%) responded that they would definitely be willing to get vaccinated (Acceptors) with the provided vaccine, with only 4.4% (n = 31) answering “no” or “not sure” (Hesitators) with respect to getting a vaccination. More than three-fourth of the physicians would recommend COVID-19 vaccination both to their family members (78.6%) and patients (81.3%). Social media (67.1%), scientific articles (65.0%), and hospital media (41.8%) were the most common sources of COVID-19 vaccination information (Table 1).

The impact of demographic characteristics on vaccine acceptance is displayed in Table 2. Acceptors tended to be older than Hesitators (Median age (IQR): 30.0 (29.0–33.0) vs 28.0 (27.0–29.0), p < 0.01). Faculty physicians had a higher proportion of Acceptors compared to fellows and residents (99.4, 98.4 and 93.1, respectively). 94% medical clerkships had a favorable attitude regarding COVID-19 immunization, despite having the lowest Acceptors percentage among the three specialty groups. Physicians who had directly cared for COVID-19 patients had a lower Acceptor rate at 93.1%, while the Acceptor rate of physicians reporting no direct care was 97.8% (p = 0.02). The proportion of participants with high self-perceived knowledge, defined as answered agree and strongly agree to the question “You have a thorough understanding of the COVID-19 vaccine”, was higher among Acceptors (p < 0.01). A greater proportion of Acceptors had relatively high and strong access to COVID-19 vaccine information (p < 0.01) (Fig. 1B1, B2). Physicians who received information from the hospital channel had a significantly higher rate of being Acceptors (p < 0.01), while those who received information primarily through social media were slightly less likely to be Acceptors (p = 0.04). It is noted that physicians receiving information from social media still showed a very high Acceptor rate.

**Table 2:** Participants characteristics by vaccine acceptance

	Hesitator	Acceptor	p value
	n = 31	n = 674	
Age, median (IQR)	28.0 (27.0–29.0)	30.0 (29.0–33.0)	< 0.01^a^
Female, n (%)^†^	18 (5.0%)	341 (95.0%)	0.47^b^
Religion, n (%)^†^			0.40^b^
Buddhism	28 (4.2%)	634 (95.8%)	
Christianity	2 (9.5%)	19 (90.5%)	
Islam	0 (0.0%)	3 (100.0%)	
Others	1 (5.3%)	18 (94.7%)	
Presence of comorbidity, n (%)^†^	4 (3.1%)	125 (96.9%)	0.63^b^
Physician role, n (%)^†^			< 0.01^b^
Resident	28 (6.9%)	378 (93.1%)	
Fellow	2 (1.6%)	126 (98.4%)	
Staff	1 (0.6%)	170 (99.4%)	
Department, n (%)^†^			0.04^b^
General/Others	10 (5.5%)	171 (94.5%)	
Medical	16 (6.0%)	249 (94.0%)	
Surgical	5 (1.9%)	254 (98.1%)	
Direct care of COVID-19 patients, n (%)^†^			< 0.01^b^
Yes	23 (6.9%)	310 (93.1%)	
No	8 (2.2%)	364 (97.8%)	
High aerosolization work settings, n (%)^†^			0.58^b^
Yes	16 (5.0%)	306 (95.0%)	
No	15 (3.9%)	368 (96.1%)	
Previous influenza vaccination, n (%)^†^			0.43^b^
Never	1 (5.0%)	19 (95.0%)	
Sometimes	6 (2.9%)	200 (97.1%)	
Yearly	24 (5.0%)	455 (95.0%)	
Preferred vaccine type, n (%)^†^			< 0.01^b^
Inactivated	1 (0.6%)	161 (99.4%)	
Viral vector	7 (5.7%)	115 (94.3%)	
mRNA	18 (7.2%)	233 (92.8%)	
Any	5 (2.9%)	165 (97.1%)	
Information sources, n (%)^†^			
Articles/Scientific journal	16 (3.5%)	442 (96.5%)	0.12^b^
TV/Newspaper	7 (2.8%)	242 (97.2%)	0.18^b^
Social media	26 (5.5%)	447 (94.5%)	0.05^b^
Hospital media	5 (1.7%)	290 (98.3%)	< 0.01^b^
Academic conference	6 (3.1%)	190 (96.9%)	0.41^b^
Friend/Family	5 (2.8%)	175 (97.2%)	0.29^b^
Others	0 (0.0%)	1 (100.0%)	0.9^b^
Willingness to recommend vaccine to family, n (%)^†^			< 0.01^c^
Yes	8 (1.4%)	546 (98.6%)	
No	10 (50.0%)	10 (50.0%)	
Not sure	13 (9.9%)	118 (90.1%)	
Willingness to recommend vaccine to patients, n (%)^†^			< 0.01^c^
Yes	11 (1.9%)	562 (98.1%)	
No	3 (21.4%)	11 (78.6%)	
Not sure	17 (14.4%)	101 (85.6%)	

^†^Percentage by row

^a^Mann–Whitney U test

^b^Fisher exact test

Roughly one-fifth (19%) of Acceptor physicians reported being hesitated to recommend the vaccine to family members and 16.6% reported a refusal to advice patients under their care to get vaccinated.

There were no differences in gender, religion, medical comorbidity, work setting with aerosol-generating procedures, previous influenza vaccination and general attitude toward COVID-19 outbreak between Acceptors and Hesitators.

The majority of 674 Acceptors (70.2%) were willing to endorse vaccination for the prevention of symptomatic illness. Acceptors also endorsed vaccination to reduce the risk during medical care (48.5%) and due to the presence of institutional support (40.2%). Reason for hesitancy by Hesitators included uncertainty of the vaccine efficacy (83.9%) and fear of adverse events (48.4%). Neurological complications (38.0%), severe allergic reaction or anaphylaxis (28.2%) and long-term side effects (24.8%) were of major concern among Hesitators (Table 3).

**Table 3:** Reasons for vaccine rejection and acceptance, along with concerns over vaccine complications

	Responder		Responder	
	n = 674		n = 31	
*Reasons behind vaccine acceptance*		*Reasons for vaccine hesitancy*		
To prevent COVID-19 infection	473 (70.2%)	Uncertain of vaccine efficacy	26 (83.9%)	
Having high risk of infection from work	327 (48.5%)	Concerns about side effects	15 (48.4%)	
Organization supports	271 (40.2%)	Low vaccine safety due to rushed development	2 (6.5%)	
Free of charges	156 (23.1%)	Low risk of infection, do not need vaccination	2 (6.5%)	
Living in close quarters with someone at high risk	154 (22.8%)	Low confidence in the vaccine technology	1 (3.2%)	
Peer pressure	4 (0.6%)	Others	0 (0.0%)	
Others	0 (0.0%)			

	Total	Yes	No/Not sure	p value^a^
	n = 705	n = 674	n = 31	
*Most concern side effects*^†^				0.66
None	3 (0.4%)	3 (0.4%)	0 (0.0%)	
Anaphylaxis	199 (28.2%)	192 (28.5%)	7 (22.6%)	
Neurologic complications	268 (38.0%)	259 (38.4%)	9 (29.0%)	
Local reaction	6 (0.9%)	6 (0.9%)	0 (0.0%)	
Systemic reaction	21 (3.0%)	20 (3.0%)	1 (3.2%)	
Long-term side effects	175 (24.8%)	163 (24.2%)	12 (38.7%)	
COVID infection	26 (3.7%)	24 (3.6%)	2 (6.5%)	
Thromboembolism/VIPIT	7 (1.0%)	7 (1.0%)	0 (0.0%)	

^†^One best choice question

^a^Fisher exact test

### Determinant of allocated COVID-19 vaccine hesitancy

Bivariate logistic regression analysis showed that several covariates including age, physician status, surgical specialty, experience of direct caring for COVID-19 patients, preference type of vaccine (mRNA, Vector), access to information, and perception of vaccine knowledge were significantly associated with being a Hesitator (Table 4). In the multivariable logistic regression model, being in the surgical department was independently associated with a lower chance of vaccine hesitation compared to general practitioners (aOR 0.24; 95% CI 0.07–0.82; p = 0.02). Physicians with access to COVID-19 vaccine information were less likely to be Hesitators (aOR 0.34; 95% CI 0.13–0.90; p = 0.03). Participants who preferred other types of vaccine rather than the inactivated virus vaccine were more likely hesitating to take the allocated vaccine with viral vector vaccine (aOR 9.96; 95% CI 1.11–81.97; p = 0.04) and mRNA vaccine preference (aOR 8.95; 95% CI 1.11–81.97; p = 0.04). Hospital-based information was significantly associated with a lower rate of being a Hesitator (aOR 0.23; 95% CI 0.08–0.68, p < 0.01). The sensitivity analysis by adding comorbidity and sex, reducing the threshold of p-value to 0.1 and 0.05 during variable selection for the multivariable model yielded similar results (Additional file 2: Table 1).

**Table 4:** Association between factors toward potential allocated COVID-19 vaccine hesitancy

Variable	Bivariate logistic regression model			Multivariable* logistic regression model		
	OR	95%CI	p value	Adjusted OR	95% CI	p value
Female	1.35	0.65–2.80	0.42	–		
Age	0.71	0.59–0.86	< 0.001	0.87	0.68–1.12	0.29
Presence of comorbidity	0.65	0.22–1.89	0.43	–		
Physician role						
Resident	*Baseline*			*Baseline*		
Fellow	0.21	0.05–0.91	0.04	0.21	0.04–1.21	0.08
Staff	0.08	0.01–0.59	0.01	0.21	0.02–2.36	0.2
Department						
General/Others	*Baseline*			*Baseline*		
Medical	1.10	0.49–2.48	0.82	1.57	0.61–4.04	0.35
Surgical	0.34	0.11–1.00	0.05	0.24	0.07–0.82	0.02
Direct care of COVID-19 patients	3.38	1.49–7.65	< 0.01	2.08	0.80–5.39	0.13
High aerosolization work settings	1.28	0.62–2.64	0.50	–		
Attitude and Information about COVID-19 (scale 1–5 as continuous variable)						
COVID-19 is a severe disease	0.75	0.52–1.09	0.13	0.75	0.48–1.19	0.23
COVID-19 impacts economy	1.02	0.56–1.85	0.96	–		
COVID-19 is preventable	0.85	0.57–1.26	0.41	–		
COVID-19 vaccine knowledge	0.32	0.20–0.52	< 0.001	1.08	0.40–2.91	0.87
Access to COVID-19 vaccine information	0.26	0.16–0.43	< 0.001	0.34	0.13–0.90	0.03
Preferred vaccine type						
Inactivated	*Baseline*			*Baseline*		
Viral vector	9.80	1.19–80.74	0.03	9.96	1.12–88.33	0.04
mRNA	12.44	1.64–94.10	0.01	8.95	1.11–81.97	0.04
Any	4.88	0.56–42.22	0.15	7.1	0.76–66.63	0.09
Information sources (yes or no)						
Articles/Scientific journal	0.56	0.27–1.15	0.12	0.88	0.35–2.19	0.78
TV/Newspaper	0.52	0.22–1.23	0.14	0.92	0.34–2.52	0.88
Social media	2.64	1.00–6.97	0.05	2.86	0.95–8.63	0.06
Hospital media	0.52	0.22–1.23	0.14	0.23	0.08–0.68	< 0.01
Academic conference	0.25	0.10–0.67	0.01	1.45	0.47–4.48	0.52
Friend/Family	0.61	0.25–1.51	0.29	–		
Others	0.55	0.21–1.45	0.23	–		

*The multivariable model included variables with a p value of < 0.2 in the bivariate model

### Attitudes toward specified vaccine technology

**Fig. 2:**
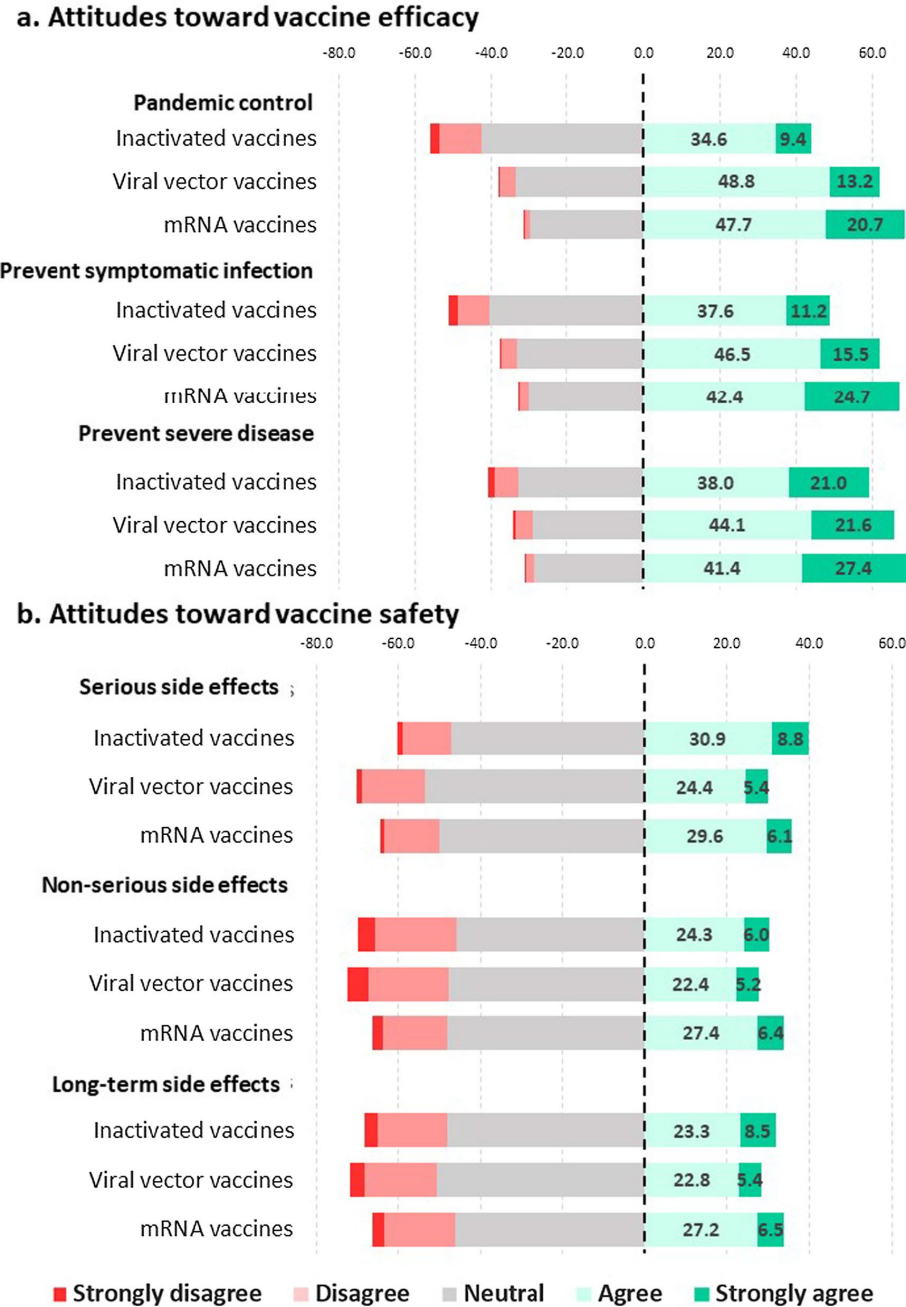
Attitude toward vaccine efficacy and safety by vaccine type

Results showed that 35.6% of physicians preferred the mRNA vaccine followed by the inactivated virus vaccine (23%) and viral vector vaccine (17.3%) with 24.1% having no vaccine preference. The mRNA vaccine was regarded as the best out of the three in terms of vaccine efficacy, followed by the viral vector vaccine and the inactivated vaccine for pandemic control, prevention of symptomatic illness and prevention of severe symptoms (Fig. 2a). Less than half of those polled believed that the inactivated vaccine would contain the COVID-19 pandemic effectively (44%) or would effectively prevent symptomatic disease (48.8%). When asked about vaccine safety in terms of non-severe side effects, serious adverse events and long-term side effects, most participants showed strongly disagree, disagree and neutral attitudes. No specific vaccine type received more than 50% strongly agree or agree response regarding safety. Physicians thought that inactivated vaccines would have less serious side effects than the other vaccines (Fig. 2b).

## Discussion

Our study is the first to measure vaccine acceptance among physicians in Asia. Representing forty percent of the doctors in one of Thailand’s largest university hospitals, the rate of vaccine acceptance at a time shortly before Thailand’s vaccine implementation was found very high at 95.6%. Only 4.4% of participants responded that they were hesitant to receive a specific brand of vaccine mandated by the Thailand National Health Authority. It should be noted that 97.1% of respondents reported also receiving previous influenza vaccinations indicating a positive attitude toward vaccination in the population. Almost all respondents felt grave impact to the personal health and the Thai economy from COVID-19, as showed in Fig. 1.

Despite a low case fatality rate (0.5%) and less than a hundred new COVID-19 cases daily at the time of the survey [1], Thai physicians' attitudes toward vaccination had already been shown to be more favorable than in studies among healthcare workers from countries more affected by COVID-19, such as Israel (78.1% in March 2020), France and Canada in (72.4% in October 2020), United States (36% in October 2020) and Columbia (90.7% in January 2021) [13, 14, 20, 21]. COVID-19 acceptance among the general Thai population was found to be between 77 and 87% in a recent survey [14, 17]. This result supports the previous research that vaccine acceptance is generally higher among healthcare workers, especially physicians [6–11]. The exceptionally high rate of vaccine acceptance found in our study was consistent with the worldwide systematic review that demonstrated highest vaccination acceptance from studies in Southeast Asian [12].

Demographic differences between COVID-19 vaccine Acceptors and Hesitators have been reported in previous studies including age, gender, previous vaccination and presence of comorbidity [13, 14]. Participants in our study reported willing to receive vaccination were older, although still quite young overall, and were faculty rather than medical residents or fellows. Not only did Acceptors have a higher perceived susceptibility to COVID-19 infection, but they were also more likely to have access to COVID-19 information, which was one of the determinants of vaccine acceptability found in this study [12]. In contrast to previous evidence, we found a lower vaccine acceptance among physicians who cared for hospitalized COVID-19 patients [12–14, 16, 17, 21]. This finding is alarming, since they are at higher risk of infection. Vaccine hesitancy rate was also higher among frontline internists and pediatricians for treating COVID-19 patients. We found that larger proportion of these doctors, in comparison to other specialist and general practitioners, were concerned about vaccine efficacy, safety and, partly, peer pressure. Overall, our results showed no differences in vaccine acceptance due to gender, religion, medical comorbidity, work setting with aerosol-generating procedures and previous influenza vaccination.

From multivariable analysis, the most important factor associated with vaccine hesitancy was a preference for alternative vaccine technology other than that allocated by the Thai Health Authority, especially for mRNA vaccine. More than one-third of physicians polled preferred the mRNA vaccine, especially in terms of vaccine efficacy and safety. At the time of the survey, only mRNA vaccines and the Aztrazeneca vaccine had shown promising results in both vaccination efficacy and safety, earning positions on the WHO SAGE Working Group emergency-use listing [22], whereas CoronaVac had not yet made its experimental data available to the public. Consistent with our finding but with a much greater extent, a study in general Indonesian population found high vaccine acceptance at 93.3% for the vaccine with 95% efficacy and only 67% for 50% efficacy vaccine [23]. These findings posed challenges to Thai policy at the time on vaccine restriction, which is limited to only CoronaVac (for age 18–60) and AstraZeneca (for age over 60). However, the situation may change after more information on safety and efficacy of CoronaVac becoming available, especially the report from WHO SAGE Working Group and its emergency-use listing.

Another factor we found associated with vaccine hesitancy was the degree of self-perceived vaccine literacy. In line with previous studies [24], we found that physicians who perceived having easier access to COVID-19 vaccine knowledge were less likely to state a hesitancy to take the vaccination, especially when information was distributed via hospital media. The social media, the major source of vaccination information among doctors in our study, has also shown trends towards contributing to the hesitation of vaccines. In order to increase positive attitudes toward vaccination among physicians, it would be reasonable to encourage them to acknowledge and appreciate accessible information from hospital newspapers, medical organizations and professional societies rather than from potentially inaccurate or propaganda sources [25]. Misinformation about vaccine effectiveness and safety, especially regarding neurological and severe adverse events, should be seen as one of the leading causes of vaccine mistrust.

With only one faculty member reluctant to be vaccinated, physicians-in-training made up most of the Hesitators. This is consistent with previous data that younger physicians were more likely to be skeptical toward vaccination [26]. Such skepticism could be explained by abundant misleading claims regarding vaccination and the anti-vaxxer movement spreading throughout mainstream media, especially on social media [27]. This concerning notion poses a challenge to medical education and training.

Despite the high rate of vaccine acceptance among doctors, as much as one-fifths of physicians were reluctant to recommend vaccination to their family members, and to the same extent, their patients. Among physicians who hesitated, the figure was even higher; 74.2% refused to vaccinate family members and 64.5% to their patients. No previous study reported such an issue. Knowingly, decreasing vaccine hesitancy among physicians should be top priority; not only they are to be among the first to receive vaccination, but they are also potentially powerful influence on public vaccination decisions [28, 29].

As cross-sectional study design, our results might not be able to predict future vaccine acceptance, as it is widely accepted that willingness to vaccinate varying through phrase of pandemic, depending on public awareness of the disease’s consequences [12]. It was conducted near the end of the second pandemic wave and right at the beginning of vaccination roll-out, when real-world data on COVID-19 vaccine performance was scarce. Moreover, selection bias from non-probabilistic snowball sampling method at a single hospital reduced generalizability of the data since our study population may not be representative of all Thai physician. In the future, probability-based sampling should be used to reproduce and strengthen the findings. Finally, the important limitation was the method for expressing vaccine acceptance. The assumption to simplify and combine “no” and “not sure” as Hesitator was made to reduce bias toward central tendency or neutral answer. Our binary representation likely led to an over-simplification of the real intention to be vaccinated.

In summary, COVID-19 vaccine acceptance is very strong among Thai doctors. However, a significant number of physicians, particularly those who hesitate, are reluctant to recommend vaccine to family members and patients. Physicians reported vaccination concerns including uncertainty of effectiveness and potential adverse reaction of the said vaccine. Barriers to vaccine acceptance were autonomy over vaccine selection and access to accurate information about the vaccine. Understanding vaccine acceptance and its determinants among physicians is critical to fostering COVID-19 vaccine adoption rate in the general population.

## Conclusion

Our research found a high rate of COVID-19 vaccine adoption among physicians, albeit slightly lower among physicians-in-training. A certain number of doctors are still reluctant to recommend the vaccine to their family and patients. Interestingly, preference of particular vaccines over the allocated option and less vaccine literacy significantly contributed to vaccine hesitancy. We believe that balancing individual autonomy within a restricted vaccination scheme, together with providing accessible, consistent, and genuine information regarding vaccines, should boost vaccine acceptance among doctors.

## Supplementary Information

**Additional file 1: Appendix A.** English-translated version of the questionnaire.

**Additional file 2: Table 1.** Sensitivity analysis for multivariate logistic regression.

## Data Availability

The datasets used and analyzed during the current study are available from the corresponding author on reasonable request.
