# Supplementary material for: COVID-19 vaccine acceptance, hesitancy, and determinants among physicians in a university-based teaching hospital in Thailand
Source: BMC Infect Dis. 2021 Nov 22;21:1174. doi: 10.1186/s12879-021-06863-5 (PMC8607407; doi:10.1186/s12879-021-06863-5)
Supplement: Supplementary file 1 — Additional file 1: Appendix A. English-translated version of the questionnaire. [file 12879_2021_6863_MOESM1_ESM.docx]

**Additional appendix A: English-translated version of the questionnaire**

**Attitudes Towards COVID-19 Vaccine Questionnaire**

**Part 1: Sociodemographic Profile**

1.1 Gender  Female  Male

1.2 Age __________ years

1.3 Religion  Buddhist  Christian  Muslim  Other __________

1.4 Physician role  Resident  Fellow  Staff

1.5 Department  Medical  Surgical  General/Others

1.6 Presence of comorbidity  No  Yes, ________________________

1.7 Have you ever provided direct care to someone tested positive for COVID-19?

 Yes  No

1.8 Does your work consist of procedure with high risk for aerosolization?

 Yes  No

1.9 How often do you receive an influenza vaccine?

 Every year  Some year  Never

**Part 2: Attitude Towards COVID-19**

| **Attitude towards COVID-19** | **Strongly disagree** | **Disagree** | **Neutral** | **Agree** | **Strongly agree** |
| --- | --- | --- | --- | --- | --- |
| COVID-19 is a severe disease |  |  |  |  |  |
| COVID-19 impacts economy |  |  |  |  |  |
| COVID-19 is preventable |  |  |  |  |  |

**Part 3: Attitude Toward receiving vaccination**

3.1 Are you willing to receive COVID-19 vaccine?

 Yes  No  Undecided

3.2 Would you recommend your family members to receive COVID-19 vaccine?

 Yes  No  Undecided

3.3 Would you recommend your patients members to receive COVID-19 vaccine?

 Yes  No  Undecided

3.4 If you have the options, which type of vaccine would you prefer?

 Inactivated virus vaccine

 Viral vector vaccine

 mRNA vaccine

 Any types of vaccine

3.5 If you answer “Yes” on question 3.1, what is/are the reason(s) for receiving COVID-19 vaccination?

 To prevent COVID-19 infection

 Having high risk of infection from work

 Organization supports

 Free of charges

 Living in close quarters with someone at high risk

 Peer pressure

 Other ____________________

3.6 If you answer “No/Undecided” on question 3.1, what is/are the reason(s) for being unsure or unwilling regarding intention to take COVID-19 vaccine?

 Uncertain of vaccine efficacy

 Concerns about side effects

 Low vaccine safety due to rushed development

 Low risk of infection, do not need vaccination

 Low confidence in the vaccine technology

 Other ____________________

3.7 What side effects concern you the most?

 None

 Anaphylaxis

 Neurologic complications

 Local reaction

 Systemic reaction

 Long-term side effects

 COVID infection

 Thromboembolism/VIPIT

**Part 4: Attitude Towards COVID vaccine types**

**4.1 Inactivated virus vaccine**

| **Attitude towards *inactivated virus vaccine*** | **Strongly disagree** | **Disagree** | **Neutral** | **Agree** | **Strongly agree** |
| --- | --- | --- | --- | --- | --- |
| You are certain of its effectiveness in |  |  |  |  |  |
| - pandemic control |  |  |  |  |  |
| - prevent symptomatic infection |  |  |  |  |  |
| - prevent severe disease |  |  |  |  |  |
| You are certain of its safety and certain that there will be |  |  |  |  |  |
| - no serious side effects |  |  |  |  |  |
| - no non-serious side effects |  |  |  |  |  |
| - no long tern side effects |  |  |  |  |  |

**4.2 Viral vector vaccine**

| **Attitude towards *viral vector vaccine*** | **Strongly disagree** | **Disagree** | **Neutral** | **Agree** | **Strongly agree** |
| --- | --- | --- | --- | --- | --- |
| You are certain of its effectiveness in |  |  |  |  |  |
| - pandemic control |  |  |  |  |  |
| - prevent symptomatic infection |  |  |  |  |  |
| - prevent severe disease |  |  |  |  |  |
| You are certain of its safety and certain that there will be |  |  |  |  |  |
| - no serious side effects |  |  |  |  |  |
| - no non-serious side effects |  |  |  |  |  |
| - no long tern side effects |  |  |  |  |  |

**4.3 mRNA vaccine**

| **Attitude towards *mRNA vaccine*** | **Strongly disagree** | **Disagree** | **Neutral** | **Agree** | **Strongly agree** |
| --- | --- | --- | --- | --- | --- |
| You are certain of its effectiveness in |  |  |  |  |  |
| - pandemic control |  |  |  |  |  |
| - prevent symptomatic infection |  |  |  |  |  |
| - prevent severe disease |  |  |  |  |  |
| You are certain of its safety and certain that there will be |  |  |  |  |  |
| - no serious side effects |  |  |  |  |  |
| - no non-serious side effects |  |  |  |  |  |
| - no long tern side effects |  |  |  |  |  |

**Part 5: COVID-19 vaccine information**

|  | **Strongly disagree** | **Disagree** | **Neutral** | **Agree** | **Strongly agree** |
| --- | --- | --- | --- | --- | --- |
| 5.1 You read and understand current knowledge regarding to COVID-19 vaccine |  |  |  |  |  |
| 5.2 You have access to information regarding to COVID-19 vaccine |  |  |  |  |  |

5.3 How do you receive information regarding to COVID-19 vaccine?

 Articles/Scientific journal  TV/Newspaper

 Social media  Hospital media

 Academic conference  Friend/Family

 Others ____________________
