# Supplementary material for: COVID-19 vaccine acceptance, hesitancy, and determinants among physicians in a university-based teaching hospital in Thailand
Source: BMC Infect Dis. 2021 Nov 22;21:1174. doi: 10.1186/s12879-021-06863-5 (PMC8607407; doi:10.1186/s12879-021-06863-5)
Supplement: Supplementary file 2 — Additional file 2: Table 1. Sensitivity analysis for multivariate logistic regression. [file 12879_2021_6863_MOESM2_ESM.docx]

**Additional table 1:** Sensitivity analysis for multivariate logistic regression

| **Variable** | **Model 1**  **sex+ comorbid + bivariate p<0.1** | | | **Model 2**  **Bivariate p < 0.1** | | | | | |
| --- | --- | --- | --- | --- | --- | --- | --- | --- | --- |
|  | **OR** | **95%CI** | **p value** | **Adjusted OR** | | **95% CI** | | **p value** | |
| **Female** | - |  |  | 1.20 | | 0.51 – 2.81 | | 0.68 | |
|  |  |  |  |  | |  | |  | |
| **Age** | 0.85 | 0.67 – 1.09 | 0.19 | 0.85 | | 0.67 - 1.09 | | 0.21 | |
|  |  |  |  |  | |  | |  | |
| **Presence of comorbidity** | - |  |  | 0.67 | | 0.20 – 2.16 | | 0.50 | |
|  |  |  |  |  | |  | |  | |
| **Physician role** |  |  |  |  | |  | |  | |
| Resident | *baseline* |  |  | *baseline* | |  | |  | |
| Fellow | 0.43 | 0.08 – 2.23 | 0.32 | 0.44 | | 0.08 – 2.27 | | 0.32 | |
| Staff | 0.27 | 0.03 – 2.75 | 0.27 | 0.27 | | 0.03 – 2.76 | | 0.27 | |
|  |  |  |  |  | |  | |  | |
| **Department** |  |  |  |  | |  | |  | |
| General/Others | *-* |  |  | *-* | |  | |  | |
| Medical | - |  |  | - | |  | |  | |
| Surgical | - |  |  | - | |  | |  | |
|  |  |  |  |  | |  | |  | |
| **Direct care of COVID-19 patients** | 1.60 | 0.66 – 3.89 | 0.30 | 1.74 | | 0.68 – 4.43 | | 0.25 | |
|  |  |  |  |  | |  | |  | |
| **High aerosolization work settings** | - |  |  | - | |  | |  | |
|  |  |  |  |  | |  | |  | |
| **Attitude and Information about COVID-19 (scale 1-5 as continuous variable)** | | | | | | | | | |
| COVID-19 is a severe disease | - |  |  | - | |  | |  | |
| COVID-19 impacts economy | - |  |  | - | |  | |  | |
| COVID-19 is preventable | - |  |  | - | |  | |  | |
| COVID-19 vaccine knowledge | 0.89 | 0.35 – 2.26 | 0.804 | 0.93 | | 0.36 – 2.42 | | 0.88 | |
| Access to COVID-19 vaccine information | 0.33 | 0.13 – 0.86 | 0.023 | 0.33 | | 0.13 – 0.86 | | 0.02 | |
|  |  |  |  |  | |  | |  | |
| **Preferred vaccine type** |  |  |  |  | |  | |  | |
| Inactivated | *baseline* |  |  |  | |  | |  | |
| Viral vector | 10.29 | 1.19-88.63 | 0.03 | 10.13 | | 1.18 – 87.08 | | 0.035 | |
| mRNA | 9.94 | 1.28-77.45 | 0.03 | 10.24 | | 1.31 – 80.01 | | 0.027 | |
| any | 6.16 | 0.69-55.34 | 0.11 | 6.355 | | 0.71 – 57.09 | | 0.099 | |
|  |  |  |  |  | |  | |  | |
| **Information sources (yes or no)** |  |  |  |  | |  | |  | |
| Articles/Scientific journal | - |  |  | - | |  | |  | |
| TV/Newspaper | - |  |  | - | |  | |  | |
| Social media | 2.10 | 0.75 – 5.86 | 0.16 | 2.19 | | 0.78 – 6.16 | | 0.82 | |
| Hospital media | - |  |  | - | |  | |  | |
| Academic conference | 1.07 | 0.39 – 2.90 | 0.90 | 1.13 | | 0.41 – 3.10 | | 0.49 | |
| Friend/Family | - |  |  | - | |  | |  | |
| Others | - |  |  | - | |  | |  | |
|  |  |  |  |  | |  | |  | |
| *The multivariable model included variables with a p value of <0.2 in the bivariate model | | | | |  | |  | |  |
